# EFFECTS OF TRANSMISSIONS OF OLDER MOTHERS’ INTERPERSONAL RELATIONSHIP QUALITY ON ADULT CHILDREN’S WELL-BEING

**DOI:** 10.1093/geroni/igac059.2340

**Published:** 2022-12-20

**Authors:** Destiny Ogle, Yifei Hou, J Jill Suitor, Megan Gilligan

**Affiliations:** Purdue University, West Lafayette, Indiana, United States; Renmin University, Beijing, Beijing, China (People's Republic); Purdue University, West Lafayette, Indiana, United States; Iowa State University, Ames, Iowa, United States

## Abstract

Prior research has rarely considered how the effects of social relationships on well-being can extend across generations. Drawing from the life-course perspective, we tested intergenerational transmission of relationship quality (with mothers and friends) as a mechanism through which mothers’ relationship quality affects adult children’s psychological well-being. We proposed that adult children bear the consequence of mothers’ relationship quality because they tend to develop interpersonal relationships similar to their mothers’ through socialization and observational learning, which in turn affects their psychological well-being. We investigated how this mechanism varied by adult children’s gender using mixed-method data collected from 693 adult children in 270 families as part of the Within-Family Difference Study. Multilevel mediation analyses revealed that transmission of mother-child closeness and tension affected adult sons’ but not daughters’ depressive symptoms. In contrast, transmission of friendship closeness decreased daughters’ but not sons’ depressive symptoms. Mother’s friendship tension affected both sons’ and daughters’ depressive symptoms, but the mechanisms differed by gender. Sons’ depressive symptoms were affected by intergenerational transmission of friendship tension. In contrast, intergenerational transmission was not the mechanism via which mothers’ friendship tension raised daughters’ depressive symptoms. Qualitative analyses revealed daughters’ well-being was affected by more diverse pathways besides intergenerational transmission of relationship quality and that these differences could be explained by gender socialization and differing meanings of social relationships for sons and daughters. These findings highlight the role of parental socialization in the mechanisms by which linked lives affect adult children’s well-being, even decades after these processes were set into motion.

